# Evacuation of a multi-loculated acute-on-chronic subdural hematoma using tandem bedside subdural evacuation port systems

**DOI:** 10.1016/j.tcr.2022.100668

**Published:** 2022-06-28

**Authors:** John K. Yue, Alexander F. Haddad, Albert S. Wang, David J. Caldwell, Gray Umbach, Anthony M. Digiorgio, Phiroz E. Tarapore, Michael C. Huang, Geoffrey T. Manley

**Affiliations:** Department of Neurosurgery, University of California San Francisco, San Francisco, CA, United States of America; Brain and Spinal Injury Center, Zuckerberg San Francisco General Hospital, San Francisco, CA, United States of America

**Keywords:** Bedside hematoma evacuation, Subdural evacuation port system, Subdural hematoma, Traumatic brain injury

## Abstract

**Background:**

Traumatic subdural hematomas (SDH) can have devastating neurologic consequences. Acute-on-chronic SDHs are more frequent in the elderly, who have increased comorbidities and perioperative risks. The subdural evacuation port system (SEPS) procedure consists of a twist drill hole connected to a single drain on suction, which can be performed at bedside to evacuate SDHs without requiring general anesthesia. However, a single SEPS can be limited due to inability to evacuate across septations between SDHs of different ages.

**Purpose:**

We present to our knowledge the first case of using tandem SEPS to evacuate a multi-loculated SDH. We discuss the technical nuances of the procedure as a treatment option for complex SDHs.

**Findings:**

An 86-year-old man with cognitive impairment and recurrent falls presented acutely after ground-level fall with worsening dysarthria and right hemiparesis. Computed tomography scan showed a 11 mm left holohemispheric mixed-density SDH with loculated acute and subacute/chronic components with 2 mm midline shift. Following two interval stability scans, the patient underwent drainage of a superficial chronic component, and a posterolateral acute/subacute component using two sequential SEPS drains at bedside in the intensive care unit. The patient's symptoms markedly improved, drains were removed, and the patient was discharged home with home health on post-procedure day 6.

**Conclusions:**

Judicious patient selection and pre-procedural planning can enable the use of tandem SEPS to evacuate multi-loculated SDHs under moderate sedation. Using multiple subdural ports to evacuate complex SDHs should be an option for proceduralists in settings where general anesthesia is not feasible.

## Introduction

Chronic subdural hematomas (SDH) are frequently encountered by neurotraumatologists with an estimated annual incidence of 1–5/100,000, predominantly in the elderly [1]. Often due to rupture of cortical bridging veins initiated by trauma, acute-on-chronic SDH can have devastating neurological consequences. Treatment options include evacuation (craniostomy, burr hole, craniotomy), prevention (middle meningeal artery embolization) [2], and observation/medical management [3]. Inflammatory pathways, angiogenesis, and coagulopathy can lead to recurrent and/or loculated chronic SDHs, which pose challenges to evacuation [4].

The effects of comorbidities and perioperative risks for SDH evacuation are amplified in elderly patients, and have espoused minimally invasive strategies. Bedside evacuation without the use of general anesthesia was first reported in 1966 [5]. The Subdural Evacuation Port System (SEPS) is a modern variant consisting of a single twist drill hole through the calvarium with puncture of dura and connection to a Jackson-Pratt-type suction drain for negative pressure evacuation of SDH, and can be performed at the bedside [6]. The use of a single SEPS drain is associated with fewer postoperative complications, lower hospitalization costs, and shorter hospital stays when compared to surgery [1].

However, single SEPS drains are often limited by view, and are at increased risk of puncturing vascularized septations in complex/loculated SDHs. We present to our knowledge the first case of using tandem SEPS to evacuate a multi-loculated SDH. We discuss the management and technical nuances of the procedure as a treatment option for complex SDHs.

## Case presentation

**Fig. 1:**
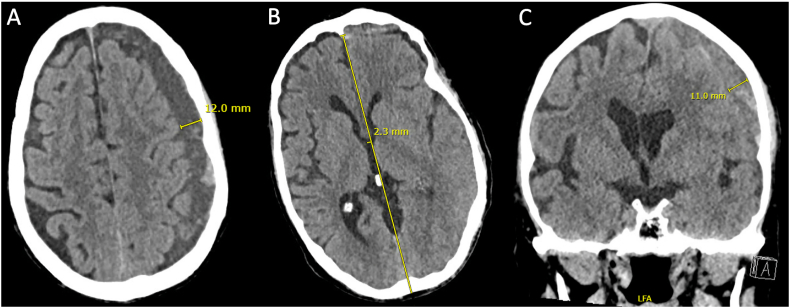
Initial Head CT, with left convexity SDH. Caption: Initial head CT with 11–12 mm left holohemispheric, multi-loculated SDH, with axial slices through the level of the corona radiata (Panel A) and coronal slice through the foramen of Monro (Panel C), with 2.3 mm of rightward midline shift (Panel B). CT = computed tomography; SDH = subdural hematoma.

An 86-year-old man not on antiplatelet or anticoagulation agents, with progressive cognitive impairment and recurrent falls presented acutely after ground-level fall with complaints of worsening dysarthria and right hemiparesis. On exam, Glasgow Coma Scale score was 14 and deficits included orientation to name and place but not year, inability to follow two-step commands, right hemibody motor strength 4/5, and right pronator drift. Head computed tomography (CT) scan showed a 11 mm left holohemispheric mixed-density SDH with loculated components, with 2 mm rightward midline shift (Fig. 1A-C). He was admitted to the intensive care unit (ICU) for neurologic exams and received a seven-day course of phenytoin for post-traumatic seizure prophylaxis. He received two interval CTs at 6 h and 18 h from admission, which showed stability of the SDH. Given his age, relative risks for anesthesia, and a stable neurologic exam, SEPS was planned and informed consent was obtained. Conscious sedation was provided by the ICU anesthesia team.

**Fig. 2:**
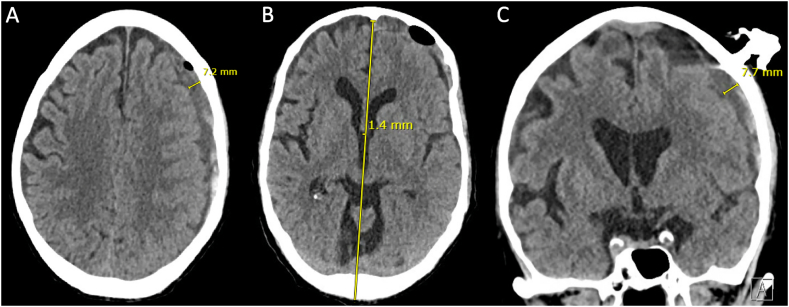
Head CT, after first SEPS. Caption: Head CT after implantation of the first SEPS 17 cm posterior to the nasion and 6 cm left of midline, with evacuation of a superficial chronic component (best seen in Panel C, intracranial and medial to the SEPS drain location). The left convexity SDH improved to 7–8 mm in thickness (Panel A and C), and midline shift improved from 2.3 mm to 1.4 mm (Panel B). CT = computed tomography; SEPS = subdural evacuation port system; SDH = subdural hematoma.

First, a thick, superficial left frontal subacute/chronic component was targeted 17 cm posterior to the nasion and 6 cm left of midline, above the superior temporal line. The SEPS twist drill hole was made and 20 mL of low-pressure, dark hygromatous fluid was evacuated. The patient's exam improved to oriented ×3. The SEPS drain was maintained to bulb suction and the patient underwent an immediate head CT which showed decrease in the thickness of the SDH to 8 mm, and decrease in midline shift to 1.4 mm. It was evident that the SEPS had evacuated a superficially-located subdural collection, and additional loculated hematomas remained especially posterolaterally (Fig. 2A-C). A second SEPS was planned 2 cm posterior and 1 cm lateral to the initial twist drill hole, over the parietal bossing.

**Fig. 3:**
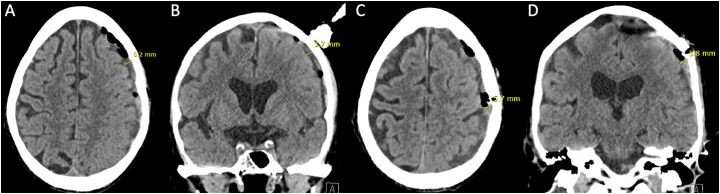
Head CT, after second SEPS. Caption: Head CT after implantation and removal of the second SEPS 2 cm posterior and 1 cm lateral to the first SEPS, with evacuation of a separate, lateral subacute SDH. Panel A and B show the more anterior component of the SDH and unchanged location of the first SEPS, with improvement of the convexity SDH to 4–6 mm. Panel C and D show the more posterior component of the SDH, which has also improved to 6 mm in thickness. CT = computed tomography; SEPS = subdural evacuation port system; SDH = subdural hematoma.

The first SEPS was placed off suction as the second twist drill hole was made, and 20 mL of thick, dark subacute SDH was evacuated. The first and second SEPS drains were alternated on and off suction over 15 min, with additional egress of 15 mL of dark blood. At this time, small amounts of subacute SDH continued to exit into the first drain, while the second drain no longer had output. To minimize multiple pressure gradients across a contiguous subdural space, the second SEPS was removed and the incision was closed with suture. A subsequent CT showed that the SDH had decreased to 6 mm in maximum thickness, and focally to 3-5 mm at the anterior and posterior twist drill hole sites, with resolution of midline shift (Fig. 3A-D). The patient's neurologic exam showed improved right hemibody strength to 4 ± 5.

**Fig. 4:**
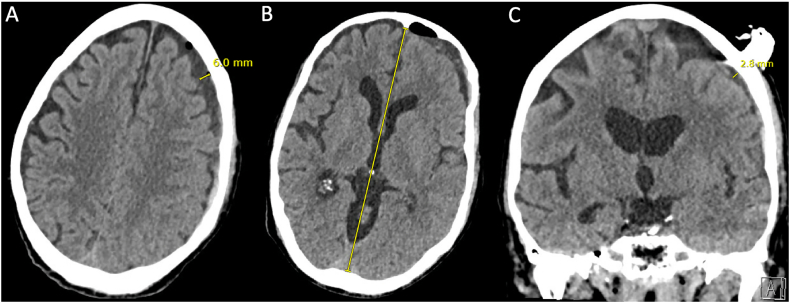
Head CT, post-procedure day 2. Caption: Head CT post-procedure day 2 after SEPS implantation. Panel A and C show further decrease in the thickness of the left convexity SDH, now 6 mm frontally and 3 mm posteriorly. Panel B shows that the prior midline shift has resolved. CT = computed tomography; SEPS = subdural evacuation port system; SDH = subdural hematoma.

Over the next 24 h, the drain output was 20 mL per 12 h shift. The patient regained the ability to follow complex commands, with motor strength 5/5 and only a subtle right pronator drift. Post-procedure day 2 CT head showed stability, with a 6 mm anterior frontal convexity (rather than holohemispheric) chronic SDH, without midline shift (Fig. 4A-C). The SEPS drain was removed on post-procedure day 2 and the exit site was closed with suture. The patient continued to improve physically and cognitively while working intensively with physical, occupational and speech therapy, and progressed to discharge home with home health on post-procedure day 6.

## Discussion

Procedures for treating SDHs of different ages risk incomplete evacuation and reaccumulation, which may lead to residual symptoms and reoperation. Across meta-analyses, the success rate of SEPS has been cited at 77–79 % with a 15–22 % recurrence rate and low morbidity of 1–2 % [1]. The efficacy of SEPS versus burr holes have been reported as similar across most studies [7], [8], Given the benefits of not requiring general anesthesia or operating room time methodological improvements of SEPS use across clinical settings is beneficial for patient outcomes. While one historical report of the SEPS technique stated the possibility of using more than one port [6], to our knowledge this is the first case report describing tandem SEPS use for the evacuation of loculated SDHs.

Our case shows that when judiciously planned based on radiographic characteristics, multiple SEPS can be used to evacuate loculated SDHs, which are traditionally difficult to evacuate [9] and beyond the capabilities of a single SEPS. Interval imaging can be used in-between SEPS placements to determine the extent and/or limits of evacuation from the first SEPS, and relative changes in morphology of other loculations subsequent to the effects of the first SEPS. Precise measurements should be taken based on anatomical landmarks prior to the placement of the second drain, as intracranial contents may have shifted. The provider should be mindful of the potential of multiple negative pressures on the intracranial space, and in our case the bulbs of each drain was set to “off suction” during placement, and with only one drain on suction at any time during active evacuation. The placement of one drain “on suction” with negative pressure, and the other “off suction”, simulates the effects of the positive pressure that encourages subdural fluid egress between two burr holes [10].

### Limitations

Our case study was performed at a well-resourced tertiary trauma center capable of acquiring urgent CT imaging within the hour, with the support of anesthesia and ICU staff. Other limitations include the favorable anatomy and physiology of the patient and the availability of multiple SEPS drains. We did not assess the effects of keeping multiple SEPS drains to suction. Our findings and these limitations await validation in larger studies across more diverse ages and SDH morphologies.

### Conclusions

Judicious patient selection and pre-procedural planning can enable successful use of tandem SEPS to evacuate multi-loculated SDHs under moderate/conscious sedation. Interval imaging can be obtained between successive SEPS to guide iterative placement of drains. The use of multiple subdural ports to evacuate complex SDHs should be an option for proceduralists in settings where general anesthesia is not feasible.

## Previous presentations

None.

## Funding

This study was unfunded.

## Declaration of competing interest

None.
